# Radical‐free hyperpolarized MRI using endogenously occurring pyruvate analogues and UV‐induced nonpersistent radicals

**DOI:** 10.1002/nbm.4584

**Published:** 2021-07-10

**Authors:** Claudia C. Zanella, Andrea Capozzi, Hikari A. I. Yoshihara, Alice Radaelli, Adèle L. C. Mackowiak, Lionel P. Arn, Rolf Gruetter, Jessica A. M. Bastiaansen

**Affiliations:** ^1^ Laboratory of Functional and Metabolic Imaging, EPFL Lausanne Switzerland; ^2^ Department of Diagnostic and Interventional Radiology Lausanne University Hospital (CHUV) and University of Lausanne (UNIL) Lausanne Switzerland

**Keywords:** cardiac metabolism, DNP, endogenous contrast agents, hyperpolarized 13C, metabolism, MRS, nonpersistent radicals, radical‐free, UV irradiation

## Abstract

It was recently demonstrated that nonpersistent radicals can be generated in frozen solutions of metabolites such as pyruvate by irradiation with UV light, enabling radical‐free dissolution dynamic nuclear polarization. Although pyruvate is endogenous, the presence of pyruvate may interfere with metabolic processes or the detection of pyruvate as a metabolic product, making it potentially unsuitable as a polarizing agent. Therefore, the aim of the current study was to characterize solutions containing endogenously occurring alternatives to pyruvate as UV‐induced nonpersistent radical precursors for in vivo hyperpolarized MRI. The metabolites alpha‐ketovalerate (*α*kV) and alpha‐ketobutyrate (*α*kB) are analogues of pyruvate and were chosen as potential radical precursors. Sample formulations containing *α*kV and *α*kB were studied with UV–visible spectroscopy, irradiated with UV light, and their nonpersistent radical yields were quantified with electron spin resonance and compared with pyruvate. The addition of ^13^C‐labeled substrates to the sample matrix altered the radical yield of the precursors. Using *α*kB increased the ^13^C‐labeled glucose liquid‐state polarization to 16.3% ± 1.3% compared with 13.3% ± 1.5% obtained with pyruvate, and 8.9% ± 2.1% with *α*kV. For [1–^13^C]butyric acid, polarization levels of 12.1% ± 1.1% for *α*kV, 12.9% ± 1.7% for *α*kB, 1.5% ± 0.2% for OX063 and 18.7% ± 0.7% for Finland trityl, were achieved. Hyperpolarized [1–^13^C]butyrate metabolism in the heart revealed label incorporation into [1–^13^C]acetylcarnitine, [1–^13^C]acetoacetate, [1–^13^C]butyrylcarnitine, [5‐^13^C]glutamate and [5‐^13^C]citrate. This study demonstrates the potential of *α*kV and *α*kB as endogenous polarizing agents for in vivo radical‐free hyperpolarized MRI. UV‐induced, nonpersistent radicals generated in endogenous metabolites enable high polarization without requiring radical filtration, thus simplifying the quality‐control tests in clinical applications.

## Graphical abstract

It was recently demonstrated that nonpersistent radicals can be generated in certain metabolites by irradiation with UV light, enabling radical‐free dissolution dynamic nuclear polarization. Although pyruvate is endogenous, an excess of additional pyruvate may perturb metabolic processes, making it potentially unsuitable as a polarizing agent when studying fatty acids or carbohydrate metabolism. Therefore, the aim of the current study was to characterize solutions containing endogenously occurring alternatives to pyruvate as UV‐induced nonpersistent radical precursors for in vivo hyperpolarized MRI.

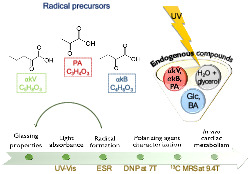

**Abbreviations used**
*α*kB: alpha‐ketobutyric acid (2‐oxobutanoic acid)
*α*kV: alpha‐ketovaleric acid (2‐oxopentanoic acid)
BA: butyric acid (butanoic acid)
DNP: dynamic nuclear polarization
ESR: electron spin resonance
FM: frequency modulation
Glc: glucose
PA: pyruvic acid (2‐oxopropanoic acid)
SNR: signal‐to‐noise ratio
TEMPOL: 1‐*λ* ^1^‐oxidanyl‐2,2,6,6‐tetramethylpiperidin‐4‐ol
TR: repetition time
UV: ultraviolet
UV–Vis: ultraviolet–visible

## 1 INTRODUCTION

Hyperpolarization via dissolution dynamic nuclear polarization (DNP) can enhance the polarization of nuclear spins by several orders of magnitude.^1^ Hyperpolarized ^13^C‐enriched probes have enabled real‐time imaging of metabolic pathways in vivo.^2^, ^3^, ^4^, ^5^, ^6^ More recently, a successful translation to human subjects was achieved,^7^ demonstrating the potential of hyperpolarized MR for several clinical applications.^8^, ^9^, ^10^, ^11^, ^12^

Hyperpolarization via DNP requires the presence of polarizing agents, in the form of free radicals, which will transfer their high spin order to the surrounding nuclei upon microwave irradiation at an appropriate frequency. Typically, these free radicals are persistent and added to the sample via chemical doping. Persistent radicals have proven to be highly efficient for dissolution DNP.^13^, ^14^ This poses a challenge for the clinical translation: free radicals may be toxic for living organisms and their presence shortens the brief measurement window of the imaging experiment.^3^, ^15^, ^16^ Employing nonpersistent radicals generated via UV light irradiation of particular precursor molecules may address this challenge. Nonpersistent radicals recombine into diamagnetic and biocompatible species at 190 K^17^ and are thereby eliminated instantly during the dissolution process, resulting in radical‐free hyperpolarized solutions. This obviates the need for filtration of the endogenous radical precursor.

Several candidates have been demonstrated as suitable precursors for UV‐induced nonpersistent radicals, namely a mixture of butanol and phenol,^18^ and several *α*‐keto acids such as pyruvic acid (PA),^19^, ^20^, ^21^, ^22^ [2‐^13^C]PA,^23^ phenylglyoxylic acid,^24^ trimethylpyruvic acid^25^ and *α*‐ketoisocaproic acid.^26^ It should be noted that trimethylpyruvic acid is not endogenous and showed great polarization capability only in its in‐house synthesized deuterated form.^25^ To date, a few in vivo studies have demonstrated the use of UV‐induced nonpersistent radicals to measure in vivo metabolic processes.^19^, ^21^, ^24^ While phenylglyoxylic acid was demonstrated to be beneficial as a polarizing agent for photosensitive metabolites,^24^ the achievable polarization levels were relatively low, at least at 3.35 T and 1.25 K. The use of PA as a polarizing agent achieved relatively high polarization levels,^19^, ^20^, ^21^, ^22^ but its presence may interfere or even compete with metabolic processes that involve or are linked to pyruvate dehydrogenase (PDH) activity or the formation of acetyl‐CoA.^6^, ^27^, ^28^, ^29^ For example, while our group has demonstrated the copolarization of [1–^13^C]butyrate, a short chain fatty acid, with [1–^13^C]pyruvate, using UV‐induced nonpersistent radicals,^21^ a prior in vivo hyperpolarized ^13^C MR study demonstrated that the metabolism of hyperpolarized [1–^13^C]butyrate was altered in the presence of PA,^6^ likely due to the acetyl‐CoA produced by increased PDH flux. For the above reasons, it is therefore of interest to find off‐the‐shelf endogenously occurring alternatives to PA for their use as nonpersistent radical precursors. Such alternatives may be advantageous for the measurement of hyperpolarized short‐ or medium‐chain fatty acid metabolism.^6^, ^30^, ^31^, ^32^, ^33^, ^34^, ^35^, ^36^ Additionally, such alternatives to PA may be beneficial for hyperpolarized MR studies, where the formation of pyruvate itself is a metabolic product of interest,^37^, ^38^, ^39^ and can therefore not be coinjected as a radical precursor.

*α*‐ketobutyrate (*α*kB) and *α*‐ketovalerate (*α*kV) are two analogues of PA that naturally occur in human blood,^40^, ^41^ but which have different biochemical properties, with their longer side chains making them poorer substrates of the enzymes PDH and lactate dehydrogenase (LDH).^41^, ^42^, ^43^, ^44^ For example, *α*kB was approximately a 50% less efficient substrate of the enzyme PDH compared with PA, while *α*kV is neither a substrate nor inhibitor of PDH,^41^ and unlikely to be a substrate for heart LDH.^43^, ^44^ Because it has been demonstrated that UV irradiation of *α*‐keto acids creates radicals,^25^ *α*kB and *α*kV may have high potential to be used as nonpersistent radical precursors.

The aim of this study was to further expand the field of UV‐induced nonpersistent radicals by characterizing the endogenous pyruvate analogues *α*kV and *α*kB following UV irradiation and to quantify their potential as endogenous polarizing agents for dissolution DNP. Effects of matrix composition on radical yields and polarization levels of ^13^C‐labeled butyric acid (BA) and glucose (Glc) were quantified and a comparison was made with PA. In a proof‐of‐concept in vivo study it was investigated whether *α*kV and *α*kB could be used to measure cardiac metabolism of ^13^C‐labeled BA.

## 2 EXPERIMENTAL

### 2.1 Sample formulation and preparation

All chemicals were ordered from Sigma‐Aldrich (Buchs, SG, Switzerland). Different sample formulations were used depending on the type of experiment.

Ultraviolet–visible (UV–Vis) spectroscopy experiments were performed at room temperature on 100 mM of *α*kV, *α*kB or PA in glycerol‐water for characterizing UV light absorption of the radical precursors.

Electron spin resonance (ESR) was used to characterize the ESR line‐shape and to quantify the concentration of the photo‐induced radicals. ESR experiments were performed with 5 M solutions of *α*kV, *α*kB or PA in glycerol: water (1:1, v/v). The ESR signal intensity was calibrated using six glycerol‐water solutions with known TEMPOL concentrations between 0 and 100 mM (Figure S1). In a second series of experiments, 2 M unlabeled Glc or 5.7 M BA was added to glycerol‐water and the amount of radical precursor was empirically optimized to obtain 40 mM of nonpersistent radicals after 200 s of UV irradiation, to ensure comparability of the DNP experiments (see the supporting information for more details on the empirical optimization procedure). Setting the target radical concentration to 40 mM was a choice made based on previous experience with broad line‐width radicals used to hyperpolarize ^13^C‐labeled nuclei at 7 T.^37^, ^45^

Hyperpolarized ^13^C MRS was performed on samples containing fully deuterated, fully ^13^C‐labeled glucose ([U‐^13^C_6_, U‐^2^H_7_]Glc) or [1–^13^C]butyric acid ([1–^13^C]BA). After optimization on samples prepared with nonlabeled compounds, the amount of radical precursor was set to 5.7 M *α*kV, 4.1 M *α*kB and 1.6 M PA for samples containing [U‐^13^C_6_, U‐^2^H_7_]Glc. Conversely, the radical precursor was set to 2.4 M *α*kV and 4.0 M *α*kB for samples containing [1–^13^C]BA. The latter were also used for in vivo measurements.

To compare liquid‐state polarization levels obtained with persistent trityl radicals, [1–^13^C]BA was hyperpolarized using OX063 by adjusting a previously published recipe^31^, ^34^ by increasing the radical concentration to 25 mM without adding a Gd‐based contrast agent. Additionally, based on the poor performance of OX063 and to compare with a matrix formulation used for the UV‐induced nonpersistent radical measurements, Finland trityl acid was added to a final concentration of 25 mM to the [1–^13^C]BA + *α*kB sample formulation (without performing UV irradiation).

### 2.2 Creating nonpersistent radicals on metabolites using UV irradiation

To create nonpersistent radicals, the sample formulations described in the previous section were sonicated and degassed at 50°C for 20 min prior to pipetting 6‐μl droplets and freezing them in liquid nitrogen to create a solid pellet. The pellets were transferred to a quartz Dewar (Magnettech, Freiberg Instruments, Germany) filled with liquid nitrogen and irradiated with a broadband UV lamp (Dymax BlueWave 200, Torrington, CT, USA) at maximum power (40 Wcm^−2^) for a maximum of 200 s using a home‐built irradiation setup.^22^

In experiments aiming to investigate the time course of radical generation for *α*kV, *α*kB or PA (*n* = 3, each precursor), frozen beads were irradiated for a set duration (i.e. 20, 45, 70, 110, 150 and 200 s), and ESR was measured at the end of each step.

### 2.3 Quantification and characterization of nonpersistent radicals

UV–Vis spectroscopy was performed to measure the light absorbance of the different radical precursors using a single beam UV‐3100PC spectrophotometer (VWR International) and a 1‐mm pathlength quartz cuvette. UV light absorption of 100 mM *α*kV, *α*kB or PA in glycerol‐water samples was measured from 280 to 600 nm in steps of 0.5. Measurements were performed at room temperature because there was no significant difference between the absorbance spectra of PA solutions acquired at room and liquid‐nitrogen temperatures.^22^

ESR was used to determine the nonpersistent radical concentration generated in the samples after UV irradiation at liquid‐nitrogen temperature. X‐band ESR was performed at 77 K as well using a MiniScope MS 400 spectrometer (Magnettech GmbH, Germany). Spectrometer parameters were chosen to ensure that saturation of the ESR signal was avoided over the entire range of radical concentrations and kept constant throughout all experiments. Parameters were set to: 20 s sweep time, 20 mT magnetic field range, 0.2 mT magnetic field modulation amplitude and 30 dB power attenuation. The ESR experiments were performed on two 6‐μl beads for each sample formulation. Subsequently, the beads were extracted from the quartz Dewar and transferred to a preweighed microcentrifuge tube, which was then weighed to determine their exact volume and correct the concentration calibration.

### 2.4 Hyperpolarization via DNP

All DNP experiments were performed in a 7‐T custom‐built polarizer. Nuclear spins were hyperpolarized using a millimeter‐wave source centered at 197 GHz, with digital control for frequency modulation (FM), a 55‐mW output power and a 1‐GHz tuning range (Elva‐1 VCOM‐06/197/1.0/55‐DD). Experiments were performed to determine the optimal hyperpolarization conditions in terms of microwave irradiation frequency and to quantify polarization build‐up times. The microwave frequency sweeps were performed with and without microwave FM to find the optimal DNP conditions and to quantify the effect of microwave FM on the enhancement. The sample cup was filled with 12 frozen UV‐irradiated beads containing [U‐^13^C, U‐^2^H]Glc. Microwave frequency‐modulated profiles were acquired at 4.2 K using a constant 40 MHz modulation amplitude and 5 kHz modulation rate for each microwave frequency step. The step size was 40 MHz for beads containing *α*kV or *α*kB, and 20 MHz for beads containing PA. For each frequency step, the sample was irradiated for 40 min and the polarization build‐up was monitored using hard 2° RF excitation pulses every 5 min. FM was not used, either for Finland trityl or for Ox063 samples.

The optimal conditions found in the previous experiments for a given set of FM parameters were used to hyperpolarize [U‐^13^C, U‐^2^H]Glc as well as [1–^13^C]BA for quantification of ^13^C polarization levels in liquid state after dissolution and for the in vivo experiments. The ^13^C‐labeled metabolites were hyperpolarized at 1.05 ± 0.02 K for 2.5 h, with frequency‐modulated (40 MHz amplitude at a rate of 5 kHz) microwave irradiation at 196.69 GHz center frequency for *α*kV and *α*kB, and at 196.65 GHz center frequency for PA. Their polarization build‐up was monitored using 2° RF excitation pulses every 5 min.

### 2.5 Dissolution DNP, hyperpolarized ^13^C MRS in phantoms and in vivo

Frozen hyperpolarized beads were dissolved using either 5.5 ml of D_2_O or a phosphate buffered saline solution for liquid‐state in vitro and in vivo experiments, respectively.^45^, ^46^ The dissolved sample was automatically transferred to a separator/infusion pump located in a 9.4‐T horizontal bore magnet (Agilent, Palo Alto, CA, USA).^47^ Hyperpolarized ^13^C MR spectra were recorded within the pump using a dual ^1^H/^13^C volume coil starting 3 s after dissolution and using a 5° RF excitation pulse with 3 s repetition time (TR). After a complete decay of the hyperpolarized magnetization, a thermal equilibrium ^13^C spectrum of the sample was acquired using a 90° RF excitation pulse, with a TR of 60 s and 64 averages. The enhancement *ϵ* was calculated as the ratio of the hyperpolarized and thermal signal peak integral, taking into account a correction for the RF excitation angle and the number of averages. The remaining ^13^C hyperpolarization after dissolution and transfer was calculated as $P=\epsilon *\tanh\left(\frac{\hbar \gamma_{C}B_{0}}{2k_{B}T}\right)$, where the hyperbolic tangent represents the ^13^C thermal equilibrium polarization at 293 K and 9.4 T.

In vivo experiments were performed on two male Wistar rats (one injection each) to demonstrate the feasibility of the novel polarizing agents to measure the cardiac metabolism of hyperpolarized [1–^13^C]BA, and to obtain preliminary information on in vivo chemical shifts. The feasibility study was conducted according to federal ethical guidelines and was approved by the local regulatory body. Anesthesia protocols and physiological monitoring were described previously.^6^, ^21^ A volume of 62‐μl [1–^13^C]BA was hyperpolarized, similar to our prior work,^6^, ^21^ with procedures described in the previous section. Frozen droplets of 10 M NaOH solution were added to the sample cup to neutralize the hyperpolarized solution during dissolution. Following an automated dissolution and transfer to the separator infusion pump,^47^ which was prefilled with 0.6 ml of phosphate buffered saline and heparin, 0.8 ml of the hyperpolarized solution was administered via a femoral vein catheter.

MR data were recorded using a custom‐made RF hybrid probe of ^1^H/^13^C‐pair surface coils placed on the chest of the animal in supine position. Correct positioning of the coil on top of the heart was ensured using gradient echo ^1^H MRI. FAST (EST)MAP shimming was performed until a ^1^H linewidth of 30 Hz was achieved. Respiratory‐gated and cardiac‐triggered unlocalized MR acquisitions were performed using a ^1^H‐decoupled (WALTZ‐16)^48^ sequence with adiabatic 30° RF excitation pulses (BIR‐4)^49^ and a TR of 3 s. Free induction decays consisting of 8258 complex data points were acquired to sample a bandwidth range of 20.5 kHz. The first spectra acquired following injection, in which metabolic products were absent, were used to confirm the resonances identified in phantom experiments (Figure S2).

### 2.6 Data processing and analysis

The ESR concentration calibration curve was obtained from fitting the second integrals of the ESR signal intensity linearly as a function of the radical concentration (Figure S1). To calculate the build‐up rate of nonpersistent radical formation, the second integral of the ESR signal intensity was corrected for bead volume variation prior to performing an intensity calibration to quantify absolute concentrations.

Following methods previously described,^22^ the radical generation time course was fit to a monoexponential function to extract the radical generation rate constant and plateau value.

Regarding the DNP sweeps, the y‐axis value at each frequency point corresponded to the polarization plateau measured at 4.2 K. The nonmodulated sweeps were normalized to 1 and the relative increase in DNP enhancement due to microwave modulation was then calculated accordingly. The microwave frequencies at which the highest polarization levels are achieved are referred to as the negative DNP maximum and the positive DNP maximum.

For each sample, the polarization time course at the best DNP condition was fit to a monoexponential function to extract the build‐up time constant and the polarization plateau, as is customary. Accordingly, each sample was irradiated for at least three time constants prior to dissolution.

Statistically significant differences between polarization levels obtained using *α*kB or *α*kV compared with PA were tested via an unpaired, two‐tail t‐test assuming equal variance, with *p* less than 0.05 considered significant.

In vivo spectra were postprocessed in VnmrJ 3.2 (Agilent, Palo Alto, CA, USA) using 20 Hz line‐broadening, baseline correction and drift correction. The signal‐to‐noise ratio (SNR) was calculated as the ratio of the highest signal intensity after phasing and the standard deviation of the noise over a region without metabolic or injected substrate resonances. All in vivo spectra where the [1–^13^C]acetylcarnitine resonance was visible were summed, corresponding to 10 consecutive spectra in the case of *α*kV, and 12 spectra in the case of *α*kB. Chemical shifts were assigned using [1–^13^C]acetylcarnitine as the reference peak resonating at 173.9 ppm and assigning all other metabolites as indicated before.^6^

## 3 RESULTS

**FIGURE 1:**
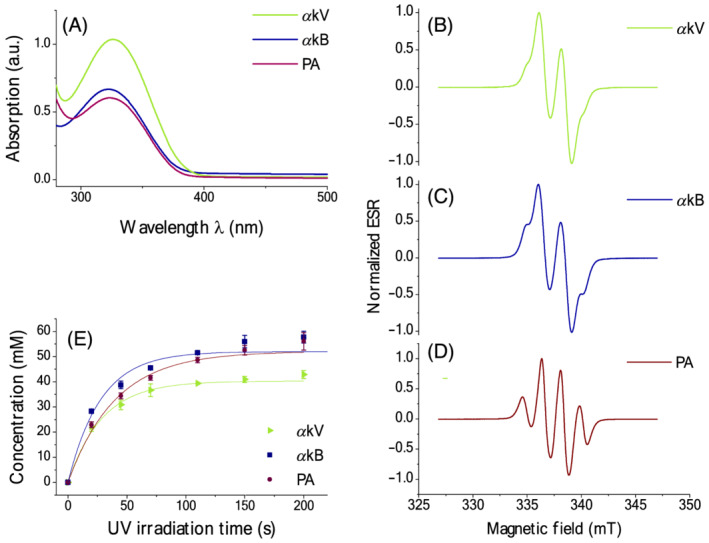
Ultraviolet–visible (UV–Vis) absorption spectra at room temperature and X‐band electron spin resonance (ESR) at 77 K. (A) UV–Vis absorption spectra of 100 mM radical precursor in glycerol‐water using a 1 mm light path showing UV‐light absorbance of alpha‐ketovaleric acid (*α*kV), alpha‐ketobutyric acid (*α*kB) and pyruvic acid (PA). (B‐D) ESR spectra of the endogenous metabolites *α*kV, *α*kB and PA at 77 K after 200 s of UV irradiation with a 40 Wcm^−2^ power UV‐light source. (E) Radical concentration build‐up curves of 5 M precursor in glycerol‐water upon UV irradiation. Table 1 lists corresponding build‐up times and maximum radical concentrations

To characterize structural changes and absorption characteristics of UV‐light irradiation on the metabolites *α*kV, *α*kB and PA, UV–Vis spectroscopy and ESR measurements were performed: UV–Vis absorption spectra showed an ~ 1.7‐fold higher absorbance for *α*kV compared with *α*kB or PA (Figure 1A). *α*kB and PA showed nearly identical absorbance maxima in the UV range of 300–400 nm (Figure 1A). Absorbance of all three metabolites peaked at around a wavelength of 320 nm.

ESR performed on frozen samples prior to UV irradiation indicated the initial absence of unpaired electron spins in the matrixes of glycerol‐water mixed with 5 M of *α*kV, *α*kB or PA. ESR spectra acquired after 200 s of UV irradiation demonstrated that free radicals were generated within the frozen samples (Figure 1B‐D). ESR spectra of *α*kV and *α*kB showed a nearly identical shape (Figure 1B,C), but were distinct from the PA spectrum (Figure 1D). The production of nonpersistent radicals as a function of UV‐irradiation time followed a near monoexponential build‐up (Figure 1E) with a characteristic time constant of 30.9 ± 5.1 s for *α*kV, 37.0 ± 5.2 s for *α*kB and 46.5 ± 1.4 s for PA (Table 1). The maximum nonpersistent radical concentrations were 41.6 ± 0.6 mM for *α*kV, 56.1 ± 2.7 mM for *α*kB and 55.0 ± 1.9 mM for PA (Figure 1E). Irradiating the samples for 200 s resulted in plateauing the radical concentration while avoiding pulverization of the beads due to excessive UV irradiation.

**TABLE 1:** UV‐induced radical build‐up times and maximum nonpersistent radical concentrations obtained after 200 s of UV irradiation in formulations containing 5 M precursor dissolved in glycerol‐water. Data were acquired at 77 K using X‐band electron spin resonance. Build‐up rates were calculated from fitting a monoexponential function to the radical concentration build‐up curves (Figure 1E). Values represent mean and standard deviation, *n* = 3

	Build‐up time (s)	Maximal concentration (mM)
***α*** **kV**	30.9 ± 5.1	41.6 ± 0.6
***α*** **kB**	37.0 ± 5.2	56.1 ± 2.7
**PA**	46.5 ± 1.4	55.0 ± 1.9

Abbreviations: *α*kB, alpha‐ketobutyric acid; *α*kV, alpha‐ketovaleric acid; PA, pyruvic acid.

**FIGURE 2:**
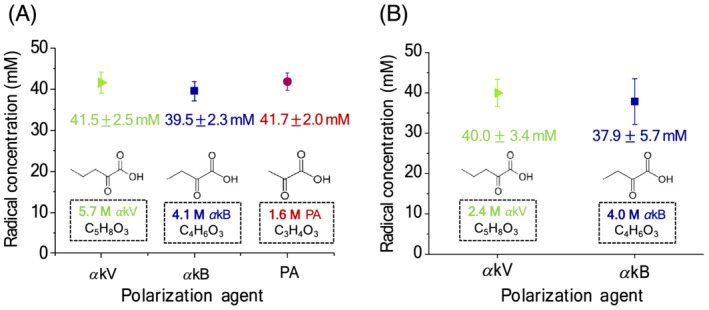
Sample formulations were optimized to obtain ~ 40 mM of nonpersistent radicals after 200 s of UV irradiation in (A) [U‐^13^C, U‐^2^H]glucose and (B) [1–^13^C]butyric acid. Underlying chemical structures are illustrated for each nonpersistent radical precursor, with the required concentrations indicated

To obtain transparent glassy beads that remained intact upon irradiation with UV light, 2 M Glc was dissolved with glycerol‐water and admixed with 5.7 M *α*kV, 4.1 M *α*kB or 1.6 M PA. These formulations yielded a nonpersistent radical concentration of 41.5 ± 2.5 mM in *α*kV, 39.5 ± 2.3 mM in *α*kB and 41.7 ± 2.0 mM in PA after 200 s of UV irradiation (Figure 2A).

**FIGURE 3:**
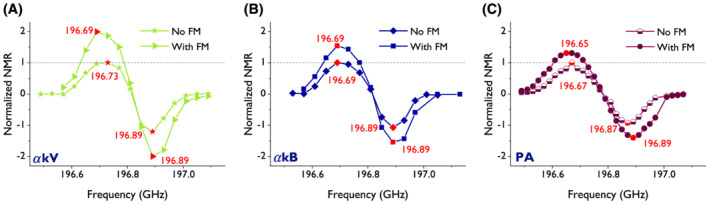
Hyperpolarized ^13^C signal as a function of microwave frequency with and without the application of frequency modulation (FM). Formulations containing [U‐^13^C, U‐^2^H]Glc in glycerol‐water were hyperpolarized at 7 T and 4.2 K. FM was set to 40 MHz amplitude at a frequency of 5 kHz. Microwave frequencies corresponding to observed dynamic nuclear polarization (DNP) maxima and minima are reported (numbers in red) in Table 2. Hyperpolarization was achieved using the UV radicals (A) alpha‐ketovaleric acid (*α*kV), (B) alpha‐ketobutyric acid (*α*kB) and (C) pyruvic acid (PA)

To assess the effect of FM of the microwave irradiation, the ^13^C nuclear polarization was measured as a function of the microwave frequency, which for the *α*kV and *α*kB sweeps showed a broadening when FM was applied. FM improved the DNP performance in terms of signal enhancement of hyperpolarized ^13^C‐labeled Glc by 100%, 50% and 30% for *α*kV, *α*kB and PA, respectively (Figure 3, Table 2). For *α*kV and *α*kB/PA, the microwave frequencies of the positive and negative DNP maxima were observed at *ν*_*max*_ = 196.69/196.65 GHz and *ν*_*min*_ = 196.89/196.89 GHz, respectively (Figure 3, Table 2).

**TABLE 2:** Microwave center frequencies at which dynamic nuclear polarization (DNP) maxima and minima occur at 7 T. Microwave frequency sweeps (Figure 3) were conducted on [U‐^13^C, U‐^2^H]Glc in glycerol‐water samples. The microwave frequency was swept using either monochromatic microwave irradiation or frequency modulation with 40 MHz modulation amplitude at a 5 kHz rate

	Microwave frequency modulation	Positive DNP maximum (GHz)	Negative DNP maximum (GHz)
***α*** **kV**	no	196.73	196.89
	yes	196.69	196.89
***α*** **kB**	no	196.69	196.89
	yes	196.96	196.89
**PA**	no	196.67	196.87
	yes	196.65	196.89

Abbreviations: *α*kB, alpha‐ketobutyric acid; *α*kV, alpha‐ketovaleric acid; PA, pyruvic acid.

To determine the build‐up rates of nuclear magnetization, the latter was measured as a function of microwave irradiation duration. The solid state build‐up times of hyperpolarized [U‐^13^C, U‐^2^H]Glc were 1.7 ± 0.5, 1.3 ± 0.5 and 1.6 ± 0.5 ks for *α*kV, *α*kB and PA, respectively (Table 3).

**TABLE 3:** Polarization build‐up time constants at 7 T, 1.05 ± 0.02 K and liquid‐state polarization levels of the C_1_, C_2 − 5_ and C_6_ resonances of [U‐^13^C_6_, U‐^2^H_7_]Glc at 9.4 T, 20°C. Liquid‐state enhancement was calculated as a ratio of hyperpolarized signal (rectangular RF excitation pulse of duration *τ* = 5 μs, RF excitation angle *α* = 5°) and thermal signal (64 averages of *α* = 90° with *τ* = 90 s, TR = 60 s). Values show mean and standard deviation over *n* = 3 datasets

*n* = 3	Build‐up time (s)	C_1_ Glc liquid‐state polarization (%)	C_2–5_ Glc liquid‐state polarization (%)	C_6_ Glc liquid‐state polarization (%)
***α*** **kV**	1.7 k ±0.5 k	9.4 ± 3.0	8.9 ± 2.1	8.0 ± 2.8
***α*** **kB**	1.3 k ± 0.5 k	16.8±0.4	16.3 ± 1.3	14.6 ± 1.7
**PA**	1.6 k ±0.5 k	13.9 ± 1.8	13.3 ± 1.5	11.5 ± 2.5
**Conditions**	7 T, 1.05 K	9.4 T, 293 K		

Abbreviations: *α*kB, alpha‐ketobutyric acid; *α*kV, alpha‐ketovaleric acid; PA, pyruvic acid.

**FIGURE 4:**
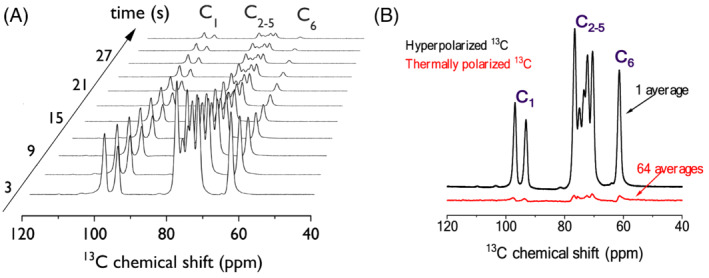
Hyperpolarized ^13^C MRS of [U‐^13^C, U‐^2^H]glucose (Glc) sample formulations. (A) Liquid‐state ^13^C signal evolution of [U‐^13^C, U‐^2^H]Glc hyperpolarized using alpha‐ketobutyric acid (*α*kB) as a polarizing agent. (B) Hyperpolarized ^13^C MR spectrum of [U‐^13^C, U‐^2^H]Glc acquired 3 s after dissolution (top) and the thermally polarized ^13^C spectrum (bottom). Acquisitions were performed at 9.4 T and 20°C

Following dissolution, the liquid‐state polarization for the C_2–5_ group of [U‐^13^C, U‐^2^H]Glc was 8.9% ± 2.1%, 16.3% ± 1.3% and 13.1% ± 1.5% for *α*kV, *α*kB and PA, respectively (Table 3, Figure 4). While [U‐^13^C, U‐^2^H]Glc hyperpolarized with *α*kB showed significantly higher liquid‐state polarization than that hyperpolarized using PA (*p* = 0.048), there was no significant difference between PA and *α*kV (*p* = 0.09). The quantification of polarization levels on the C_1_ and C_6_ resonances of Glc did not alter the results (Table 3).

**FIGURE 5:**
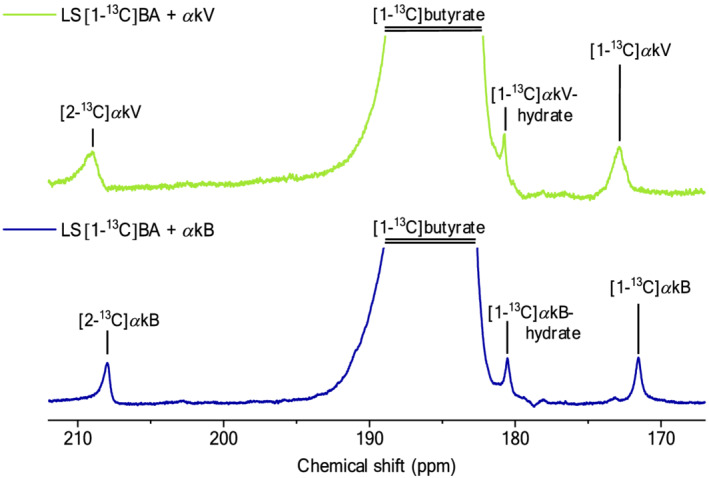
Liquid‐state ^13^C MR spectra of [1–^13^C]butyrate hyperpolarized using alpha‐ketovaleric acid (*α*kV) (top) and alpha‐ketobutyric acid (*α*kB) (bottom) displaying the ^13^C‐labeled substrate and precursor resonances. Spectra were acquired 3 s postdissolution and were line‐broadened with 2 Hz. Solutions were acidic because pH neutralization was not performed in these experiments

The solid state build‐up times of hyperpolarized [1–^13^C]butyrate were 2.1 ± 0.3 and 3.3 ± 0.4 ks when hyperpolarized using *α*kV and *α*kB, respectively, and 0.8 ± 0.3 and 1.7 ± 0.3 ks for OX063 and Finland trityl radicals, respectively. Liquid‐state enhancement over thermal polarization at 9.4 T was 14.6 k ± 1.4 k and 15.6 k ± 1.7 k for the *α*kV and *α*kB samples, respectively, which translated to liquid‐state polarization of 12.1% ± 1.1% and 12.9% ± 1.7% (Table 4). The liquid‐state polarization for OX063 and Finland trityl was 1.5% ± 0.2% and 18.7% ± 0.7%, respectively. In these experiments, the natural abundance ^13^C resonances of *α*kV and *α*kB could be identified as [1–^13^C]*α*kV (at 172.9 ppm), [1–^13^C]*α*kV‐hydrate (at 180.7 ppm), [2‐^13^C]*α*kV (at 209.1 ppm), [1–^13^C]*α*kB (at 171.9 ppm), [1–^13^C]*α*kB‐hydrate (at 180.6 ppm) and [2‐^13^C]*α*kB (at 208.4 ppm) (Figure 5). Note that pH was not neutralized in these experiments, and the chemical shifts were different in the pH‐neutralized in vivo experiments (Table 5).

**TABLE 4:** Solid state build‐up times at 7 T, 1.05 K are reported for [1–^13^C]butyrate samples containing alpha‐ketovaleric acid (*α*kV) or alpha‐ketobutyric acid (*α*kB) as nonpersistent radical precursors, and OX063 and Finland trityl as persistent radicals. Room‐temperature liquid‐state enhancements and liquid‐state polarization were calculated after sample dissolution and transfer to a 9.4‐T MR scanner. Mean and average values are obtained from *n* datasets. Exemplary spectra for the nonpersistent radical samples are shown in Figure 5

Radical	Build‐up time (s)	*n*	Liquid‐state enhancement	BA liquid‐state polarization (%)	*n*
***α*** **kV**	2.1 k ± 0.3 k	4	14.6 k ± 1.4 k	12.1 ± 1.1	3
***α*** **kB**	3.3 k ± 0.4 k	5	15.6 k ± 1.7 k	12.9 ± 1.7	3
**Trityl OX063**	0.8 k ± 0.3 k	3	1.8 k ± 0.2 k	1.5 ± 0.2	3
**Finland Trityl**	1.7 k ± 0.3 k	3	22.7 k ± 0.8 k	18.7 ± 0.7	3
**Conditions**	solid state: 7 T, 1.05 K		liquid state: 9.4 T, 293 K		

**TABLE 5:** Chemical shifts of observed metabolites in the heart after injection of pH‐neutralized hyperpolarized [1–^13^C]butyrate in vivo using either alpha‐ketovaleric acid (*α*kV) or alpha‐ketobutyric acid (*α*kB) as a polarizing agent (in vivo spectra are displayed in Figure 6). [1–^13^C]acetylcarnitine was used as the reference peak (*) and assigned to 173.9 ppm. The resonance of natural abundance [1–^13^C]*α*kV‐hydrate was not detected in these experiments (see also Figure S2)

Metabolite	Chemical shift (ppm)
natural abundance [2‐^13^C]*α*kB	209.3
natural abundance [2‐^13^C]*α*kV	208.7
[1–^13^C]butyrate	184.8
[5‐^13^C]glutamate	182.4
[5‐^13^C]citrate	179.8
natural abundance [1–^13^C]*α*kB‐hydrate	178.1
[1–^13^C]butyrylcarnitine	176.4
[1–^13^C]acetoacetate	176.0
[1–^13^C]acetylcarnitine	173.9*
natural abundance [1–^13^C]*α*kB	172.1
natural abundance [1–^13^C]*α*kV	172.0

**FIGURE 6:**
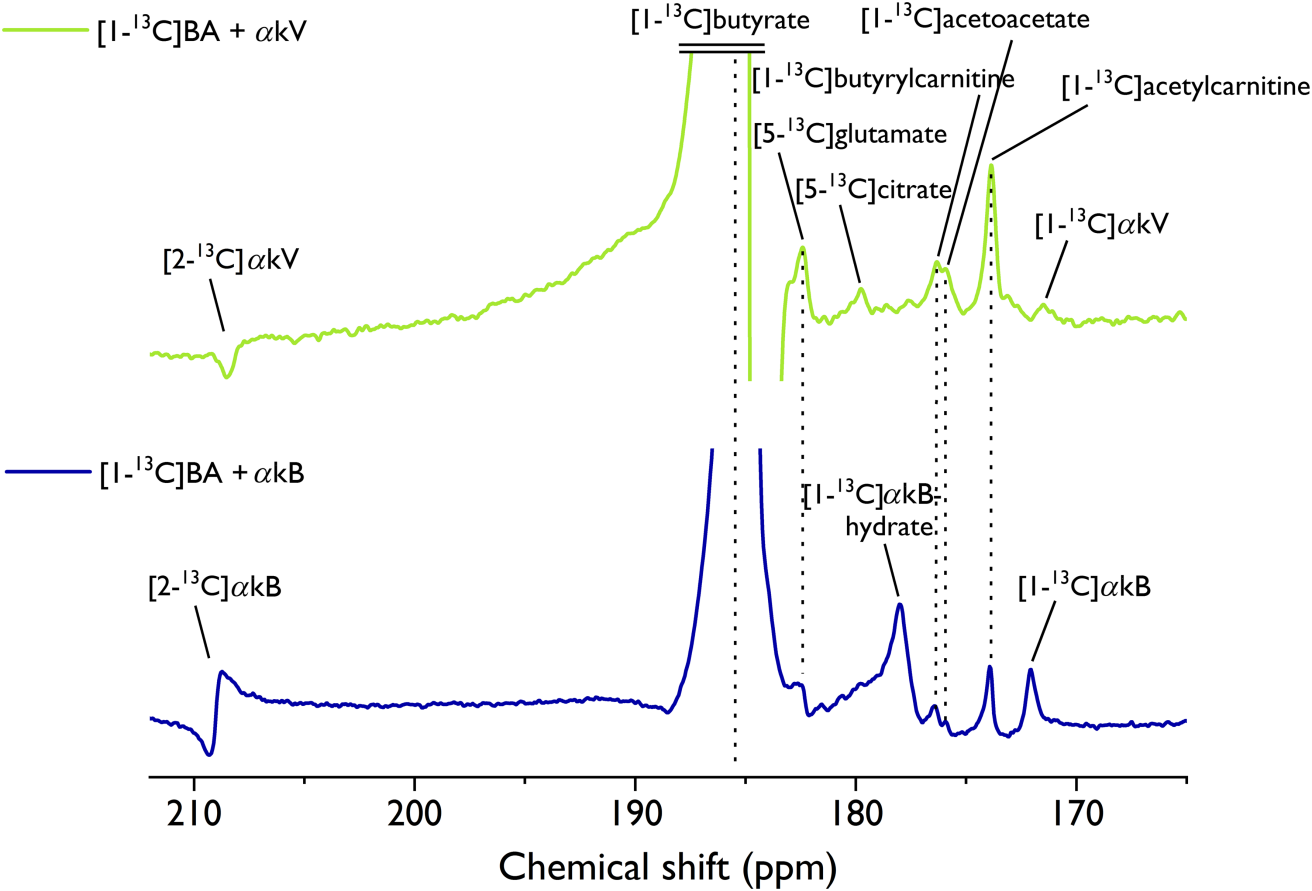
In vivo spectra of cardiac metabolism in two male Wistar rats following the injection of a radical‐free hyperpolarized [1–^13^C]butyrate solution. Hyperpolarization via dynamic nuclear polarization (DNP) was performed at 7 T, 1.05 K using the UV‐induced nonpersistent radicals alpha‐ketovaleric acid (*α*kV) (top) and alpha‐ketobutyric acid (*α*kB) (bottom). Spectra where [1–^13^C]acetylcarnitine resonances were visible were summed and line‐broadened by a factor of 20. Therefore, spectra acquired at 24–51 s (*α*kV) and 18–51 s postdissolution (*α*kB) were summed. Cardiac metabolism resulted in the detection of [1–^13^C]acetylcarnitine, [1–^13^C]acetoacetate, [1–^13^C]butyrylcarnitine and [5‐^13^C]glutamate. [5‐^13^C]citrate could not be detected in the *α*kB experiment, due to the proximity and phase of the *α*kB‐hydrate resonance. The natural abundance resonances of the injected precursors *α*kB and *α*kV were also identified. The butyrate resonance in the experiment with *α*kV was distorted by motion or flow. Similarly, the [2‐13C]*α*KB peak could not be brought into phase with the other resonances using first‐order correction

To assess the potential to measure cardiac metabolism, hyperpolarized [1–^13^C]butyrate was injected in male Wistar rats. For the first few seconds after injection of the hyperpolarized solution, metabolic products were not observable, and the detected ^13^C resonances could be identified as [1–^13^C]butyrate (at 184.8 ppm), and natural abundance resonances of [1–^13^C]*α*kV (at 172.0 ppm), [2‐^13^C]*α*kV (at 208.7 ppm), [1–^13^C]*α*kB‐hydrate (at 178.1 ppm), [1–^13^C]*α*kB (at 172.1 ppm) and [2‐^13^C]*α*kB (at 209.3 ppm). The resonance of [1–^13^C]*α*kV‐hydrate was not detected in vivo, further evidenced by its absence immediately after injection (Figure S2). Maximum in vivo SNR on ^13^C BA was observed 12 s after dissolution, with an SNR of 1370 for *α*kV and 1780 for *α*kB. Cardiac metabolism of hyperpolarized [1–^13^C]BA (Figure 6, Table 5) resulted in ^13^C‐labeling of [1–^13^C]acetylcarnitine (173.9 ppm), [1–^13^C]acetoacetate (176.0 ppm), [1–^13^C]butyrylcarnitine (176.4 ppm), [5‐^13^C]glutamate (182.4 ppm) and [5‐^13^C]citrate (179.8 ppm). Due to the shoulder of the nearby *α*kB‐hydrate resonance, [5‐^13^C]citrate could not be detected in the *α*kB experiment.

## 4 DISCUSSION

This study shows that *α*kV and *α*kB can be used to generate nonpersistent radicals for hyperpolarizing ^13^C‐labeled substrates, expanding the field of research on radical‐free dissolution DNP performed on mixtures containing only endogenously occurring substances. Their potential as polarizing agents for in vivo metabolic studies was also shown in proof‐of‐concept experiments performed in the heart.

Although UV‐irradiated *α*kV and *α*kB demonstrated virtually identical ESR line‐shapes, their reaction to UV light visibly differed (Figure 1). This was not only seen in the different UV–Vis absorption at 100 mM but also in their different radical yield at 5 M. The likely reason why *α*kV shows considerably higher absorption compared with *α*kB and PA, but lower maximum radical concentration, can be ascribed to the fact that UV light cannot efficiently penetrate through the bead volume, with a high precursor concentration. Adding Glc or BA further changed the reaction of each compound to UV light such that a specific sample formulation was required to achieve the targeted nonpersistent radical concentration of 40 mM. As has been observed before,^22^ the relationship between nonpersistent radical yield and the concentration of the precursor was nonlinear. This posed a challenge during sample formulation optimization. Even although *α*kV, *α*kB and PA differ from each other by additional methylene units on their aliphatic side chain, the results from our experiments illustrate that sample formulation requires a careful optimization in terms of UV‐induced radical yield and polarization level for each ^13^C‐labeled metabolic substrate, which is an empirical and nontrivial process. Because the current work focused on the feasibility of *α*kV and *α*kB as nonpersistent radical precursors, optimization of the sample formulation did not go beyond obtaining the targeted concentration of 40 mM nonpersistent radicals, resulting in relatively large concentrations of precursor compared with PA in the ^13^C‐labeled sample formulations. It is important to point out that the samples were not specifically optimized to maximize radical yield, to minimize precursor concentrations or to maximize achievable polarization levels, which is a limitation of our study. The empirical approach of optimizing sample formulations, plus the observation that radical quantum yields were comparable in glycerol‐water mixtures (Figure 1), show that there is significant room for improvement. Our experience with UV‐irradiated nonpersistent radical precursors and published work^21^, ^22^ indicate that precursor volumes can be reduced when boosting radical yield or UV‐light penetration, which may be achieved by using different glassing agents such as ethanol, by deuteration of the radical precursors, by changing the relative ratios of precursor to glassing agent, by adapting the UV‐irradiation time, or by changing the bead size.

The nuclear polarization levels increased when applying microwave FM, which is a consequence of the relatively broad radical ESR spectrum and short electron *T*_*1*_ characterizing the UV‐radical family (~ 100 ms).^50^, ^51^, ^52^ By contrast, microwave FM has little effect on narrow linewidth radicals and radicals with long electron *T*_*1*_ such as OX063 at 6.7 T.^53^ Similarly, for polarizer systems operating at low field (~3.35 T), microwave FM may be less effective because of longer radical T1e and more narrow spectral width (less g‐anisotropy). Microwave FM is a feature that is available on commercially available polarizers working at high field (e.g. the Spin Aligner polarizer; Polarize, Copenhagen, Denmark). Polarization levels in the current study were of the order of 9% to 16% for Glc and 13% for BA, close to the polarization of 18.7% measured when adding Finland trityl to a non‐UV–irradiated *α*kB BA mixture. The recipe for OX063 did not perform as expected on our system based on the low polarization (~2%) that was obtained. This can be a consequence of the apolar nature of the BA sample formulation. Indeed, Ox063 is very soluble in polar solvents such as water (log P of −0.5 and −1.38, respectively). Differently, BA has a log P of 0.79. Most likely, OX063 in the presence of BA made a suspension rather than a solution. The opposite reasoning applies to Finland trityl. In previous work using similar experimental conditions, UV‐irradiated metabolite mixtures containing ^13^C‐labeled PA and BA were hyperpolarized to significantly lower levels (i.e. 3.3% ± 0.5%−5.2% ± 0.5% for ^13^C BA).^21^ The latter was a consequence of a different UV‐light source, yielding a third of the radical concentration measured in this study, and the absence of microwave FM.^21^ In the current study, a broadband UV‐light source was used, delivering 40 times more power, and glycerol‐water was added to the mixtures to improve the DNP sample matrix. Although performed under different experimental conditions and using different methods to calculate the liquid‐state polarization, previous studies reported polarization levels of between 22.2% ± 2.1%^54^ and 30.1% ± 1.8%^25^ for Glc and of between 7% ± 2%^31^ and 28% ± 4%^34^ for BA. The current results are within a similar range and thus are promising. Although a dedicated optimization in terms of polarization level was not performed in the current study, polarization levels may be further increased by optimization of the sample formulation (i.e. components, concentrations and bead size), or by a detailed investigation of the microwave FM. However, previously reported improvements in DNP performance may conflict with the UV performance and nonpersistent radical generation: for example, using dimethyl sulfoxide as the glassing agent increased the DNP performance by 18‐fold when polarizing ^13^C‐labeled BA.^34^ However, UV–Vis spectroscopy and ESR measurements have shown the photosensitive nature of dimethyl sulfoxide and its unsuitability as a glassing agent for UV‐induced nonpersistent radical formulations using a broadband UV source.^24^

Hyperpolarized BA has been used before as a probe to study short‐chain fatty acid cardiac metabolism,^6^, ^21^, ^31^, ^34^, ^35^ with different results in terms of the amount of observed metabolites. In the current study, hyperpolarization of BA with *α*kV and *α*kB reached sufficient SNR to observe cardiac metabolism, with a similar metabolic profile observed in previous studies using persistent radicals at high magnetic field.^6^, ^31^ High magnetic field allows for a better spectral resolution and thus may facilitate the resolved detection of metabolites such as ^13^C‐labeled citrate and glutamate. However, *B*_*0*_ inhomogeneities also increase, which may complicate shimming procedures, especially around the moving heart, which may result in distorted line shapes and difficult‐to‐phase spectra. In the current study, the citrate resonance could not be reliably detected due to the shoulder of the neighboring [1–^13^C]*α*kB‐hydrate resonance. Conversely, the absence of [1–^13^C]*α*kV‐hydrate (Figure 6) enabled citrate detection when using *α*kV. Note that the relative amount of *α*kV in the BA sample formulation was much smaller compared with the amount of *α*kB, which contributed to increased signal intensities of natural abundance *α*kB resonances compared with those of *α*kV. This can be appreciated when observing the relative signal ratio of [1–^13^C]*α*kV or [1–^13^C]*α*kB with [1–^13^C]acetylcarnitine (Figure 6). Furthermore, the detection of [1–^13^C]*α*kV‐hydrate in the non‐pH–neutralized liquid‐state experiments compared with its absence in the pH‐neutralized in vivo experiment, as well as the increased signal intensity of the [1–^13^C]*α*kB‐hydrate resonance in vivo compared with the non‐pH–neutralized liquid‐state experiment, demonstrate the sensitivity of the proposed polarizing agents to pH and illustrate the importance of pH optimization.

The use of endogenous *α*kV and *α*kB is a potential alternative to PA as nonpersistent radical precursors. Based on the location of their natural abundance ^13^C resonances, the potential detection of pyruvate as metabolic product would not be disturbed. It is, however, a limitation of the current study that interferences of *α*kV and *α*kB with in vivo metabolic processes were not investigated. Reportedly, PDH, as well as LDH, have reduced or even absent affinities for *α*kB and *α*kV, respectively.^41^, ^42^, ^43^, ^44^ This was partially confirmed in recent in vivo experiments using hyperpolarized ^13^C‐labeled *α*kB that showed a decreased ^13^C‐labeling of bicarbonate compared with PA.^33^ Also, *α*kB is converted by PDH to propionyl‐CoA, avoiding any potential competing perturbation of the acetyl‐CoA pool, as seen with PA.^6^ In the former study the in vitro LDH response to ^13^C‐labeled *α*kB varied depending on the LDH isoform.^33^ While *α*kV is not anticipated to be appreciably converted by PDH and LDH,^41^, ^43^, ^44^ determining their in vivo response to hyperpolarized ^13^C‐labeled *α*kV, and elucidating any potential metabolic interference by it or *α*kB, including dose dependencies, remains to be established. Based on the aforementioned evidence of *α*kV not being a significant substrate of either LDH or PDH, and the lower *α*kV hydrate signal observed in our study, we hypothesize that *α*kV may be the preferred choice over *α*kB of nonpersistent radical precursor.

Nevertheless, radical‐free dissolution DNP via the use of endogenous nonpersistent radicals provides a benefit at low cost and may increase the duration of the hyperpolarized state by avoiding one filtration step in clinical applications. In addition, UV‐induced radicals of PA quench with increasing temperature^18^ and recombine at a threshold of 190 K,^17^ providing an additional benefit of UV‐induced radicals in *α*kV and *α*kB to produce transportable hyperpolarized ^13^C‐labeled substrates.

## 5 CONCLUSION

We conclude that the endogenous *α*‐keto acids *α*kV and *α*kB can be used as efficient endogenous nonpersistent radicals following irradiation with UV light, achieving similar or higher ^13^C polarization compared with PA and thus are potential candidates for translational clinical hyperpolarized MRI, enabling high polarization without requiring radical filtration. Cardiac metabolism of ^13^C‐labeled butyrate hyperpolarized with *α*kV or *α*kB demonstrated label propagation in a wide range of metabolites, demonstrating their potential as endogenous polarizing agents for in vivo radical‐free hyperpolarized MRI.

## Supporting information


**Figure S1.** Concentration calibration curve of TEMPOL in glycerol‐water (v/v 1:1) and linear fit (R^2^ = 0.999, red) for n = 4 data sets. Data was acquired with X‐band ESR at 77 K and subsequently used to quantify concentrations of UV‐induced radicals.
**Figure S2.** In vivo spectra acquired immediately after injection depict the injected ^*13*^C labeled substrate and the natural abundance ^*13*^C resonances of the polarizing agent *α*kV (top) and *α*kB (bottom). Metabolic products are absent in these spectra. Visible resonances are [1–^*13*^C]butyrate, [1–^*13*^C]*α*kV, [2‐^*13*^C]*α*kV, [1–^*13*^C]*α*kB‐hydrate, [1–^*13*^C]*α*kB and [2‐^*13*^C]*α*kB. The resonance of [1–^*13*^C]*α*kV‐hydrate could not be detected.

## References

[1] Ardenkjær‐Larsen JH , Fridlund B , Gram A , et al. Increase in signal‐to‐noise ratio of > 10,000 times in liquid‐state NMR. Proc Natl Acad Sci. 2003;100(18):10158‐10163. 12930897 10.1073/pnas.1733835100 PMC193532

[2] Golman K , Thaning M . Real‐time metabolic imaging. Proc Natl Acad Sci. 2006;103(30):11270‐11275. 16837573 10.1073/pnas.0601319103 PMC1544077

[3] Brindle KM . Imaging metabolism with hyperpolarized 13C‐labeled cell substrates. J Am Chem Soc. 2015;137(20):6418‐6427. 25950268 10.1021/jacs.5b03300

[4] Yoshihara HA , Bastiaansen JA , Berthonneche C , Comment A , Schwitter J . An intact small animal model of myocardial ischemia‐reperfusion: Characterization of metabolic changes by hyperpolarized 13C MR spectroscopy. Am J Physiol Heart Circ Physiol. 2015;309(12):H2058‐H2066. 26453328 10.1152/ajpheart.00376.2015

[5] Comment A . Dissolution DNP for in vivo preclinical studies. J Magn Reson. 2016;264:39‐48. 26920829 10.1016/j.jmr.2015.12.027

[6] Bastiaansen JA , Merritt ME , Comment A . Measuring changes in substrate utilization in the myocardium in response to fasting using hyperpolarized [1‐13 C] butyrate and [1‐13 C] pyruvate. Sci Rep. 2016;6(1):1‐11. 27150735 10.1038/srep25573 PMC4858671

[7] Nelson SJ , Kurhanewicz J , Vigneron DB , et al. Metabolic imaging of patients with prostate cancer using hyperpolarized [1–13C] pyruvate. Sci Transl Med. 2013;5(198):198ra108. https://pubmed.ncbi.nlm.nih.gov/23946197/ 10.1126/scitranslmed.3006070 PMC4201045 23946197

[8] Malloy CR , Merritt ME , Dean SA . Could 13C MRI assist clinical decision‐making for patients with heart disease? NMR Biomed. 2011;24(8):973‐979. 21608058 10.1002/nbm.1718 PMC3174329

[9] Cunningham CH , Lau JY , Chen AP , et al. Hyperpolarized 13C metabolic MRI of the human heart: initial experience. Circ Res. 2016;119(11):1177‐1182. 27635086 10.1161/CIRCRESAHA.116.309769 PMC5102279

[10] Aggarwal R , Vigneron DB , Kurhanewicz J . Hyperpolarized 1‐[13C]‐pyruvate magnetic resonance imaging detects an early metabolic response to androgen ablation therapy in prostate cancer. Eur Urol. 2017;72(6):1028‐1029. 28765011 10.1016/j.eururo.2017.07.022 PMC5723206

[11] Miloushev VZ , Granlund KL , Boltyanskiy R , et al. Metabolic imaging of the human brain with hyperpolarized 13C pyruvate demonstrates 13C lactate production in brain tumor patients. Cancer Res. 2018;78(14):3755‐3760. 29769199 10.1158/0008-5472.CAN-18-0221 PMC6050093

[12] Cleveland ZI , Bdaiwi AS , Hossain MM , et al. Hyperpolarized 129Xe diffusion MRI in cystic fibrosis lung disease: evidence for pathological alveolar enlargement. Abstract A108. Am J Respir Crit Care Med. 2019;199:A2568‐A2568.

[13] Lumata L , Merritt ME , Malloy CR , Sherry AD , Kovacs Z . Impact of Gd3+ on DNP of [1‐13C] pyruvate doped with trityl OX063, BDPA, or 4‐oxo‐TEMPO. J Phys Chem A. 2012;116(21):5129‐5138. 22571288 10.1021/jp302399f PMC3366031

[14] Yoshihara HA , Can E , Karlsson M , Lerche MH , Schwitter J , Comment A . High‐field dissolution dynamic nuclear polarization of [1‐13 C] pyruvic acid. Phys Chem Chem Phys. 2016;18(18):12409‐12413. 27093499 10.1039/c6cp00589f

[15] Ardenkjaer‐Larsen JH . On the present and future of dissolution‐DNP. J Magn Reson. 2016;264:3‐12. 26920825 10.1016/j.jmr.2016.01.015

[16] Miéville P , Ahuja P , Sarkar R , et al. Scavenging free radicals to preserve enhancement and extend relaxation times in NMR using dynamic nuclear polarization. Angew Chem Int Ed. 2010;49(35):6182‐6185. 10.1002/anie.201000934 20665608

[17] Capozzi A , Cheng T , Boero G , Roussel C , Comment A . Thermal annihilation of photo‐induced radicals following dynamic nuclear polarization to produce transportable frozen hyperpolarized 13 C‐substrates. Nat Commun. 2017;8(1):1‐7. 28569840 10.1038/ncomms15757 PMC5461505

[18] Kumada T , Noda Y , Hashimoto T , Koizumi S . Dynamic nuclear polarization study of UV‐irradiated butanol for hyperpolarized liquid NMR. J Magn Reson. 2009;201(2):115‐120. 19781965 10.1016/j.jmr.2009.08.011

[19] Eichhorn TR , Takado Y , Salameh N , et al. Hyperpolarization without persistent radicals for in vivo real‐time metabolic imaging. Proc Natl Acad Sci. 2013;110(45):18064‐18069. 24145405 10.1073/pnas.1314928110 PMC3831441

[20] Capozzi A , Hyacinthe J‐N , Cheng T , et al. Photoinduced nonpersistent radicals as polarizing agents for X‐nuclei dissolution dynamic nuclear polarization. J Phys Chem C. 2015;119(39):22632‐22639.

[21] Bastiaansen JA , Yoshihara HA , Capozzi A , et al. Probing cardiac metabolism by hyperpolarized 13 C MR using an exclusively endogenous substrate mixture and photo‐induced nonpersistent radicals. Magn Reson Med. 2018;79(5):2451‐2459. 29411415 10.1002/mrm.27122 PMC5821575

[22] Capozzi A , Karlsson M , Petersen JR , Lerche MH , Ardenkjaer‐Larsen JH . Liquid‐state 13C polarization of 30% through photoinduced nonpersistent radicals. J Phys Chem C. 2018;122(13):7432‐7443.

[23] Pinon AC , Capozzi A , Ardenkjær‐Larsen JH . Hyperpolarized water through dissolution dynamic nuclear polarization with UV‐generated radicals. Commun Chem. 2020;3(1):1‐9. 10.1038/s42004-020-0301-6 PMC9814647 36703471

[24] Marco‐Rius I , Cheng T , Gaunt AP , et al. Photogenerated radical in phenylglyoxylic acid for in vivo hyperpolarized 13C MR with photosensitive metabolic substrates. J Am Chem Soc. 2018;140(43):14455‐14463. 30346733 10.1021/jacs.8b09326 PMC6217999

[25] Capozzi A , Patel S , Gunnarsson CP , et al. Efficient hyperpolarization of U‐13C‐glucose using narrow‐line UV‐generated labile free radicals. Angew Chem Int Ed. 2019;58(5):1334‐1339. 10.1002/anie.201810522 PMC6531289 30515929

[26] Patel S , Pinon AC , Lerche MH , Karlsson M , Capozzi A , Ardenkjær‐Larsen JH . UV‐irradiated 2‐keto‐(1‐13C)isocaproic acid for high‐performance 13C hyperpolarized MR. J Phys Chem C. 2020;124(43):23859‐23866.

[27] Olson RE . Effect of pyruvate and acetoacetate on the metabolism of fatty acids by the perfused rat heart. Nature. 1962;195(4841):597‐599. 14481944 10.1038/195597b0

[28] Olson MS , Dennis SC , DeBuysere MS , Padma A . The regulation of pyruvate dehydrogenase in the isolated perfused rat heart. J Biol Chem. 1978;253(20):7369‐7375. 701258

[29] Moreno KX , Sabelhaus SM , Merritt ME , Sherry AD , Malloy CR . Competition of pyruvate with physiological substrates for oxidation by the heart: implications for studies with hyperpolarized [1‐13C] pyruvate. Am J Physiol Heart Circ Physiol. 2010;298(5):H1556‐H1564. 20207817 10.1152/ajpheart.00656.2009 PMC2867437

[30] Bastiaansen JA , Cheng T , Mishkovsky M , Duarte JM , Comment A , Gruetter R . In vivo enzymatic activity of acetylCoA synthetase in skeletal muscle revealed by 13C turnover from hyperpolarized [1–13C] acetate to [1–13C] acetylcarnitine. Biochim Biophys Acta Gen Subj. 2013;1830(8):4171‐4178. 10.1016/j.bbagen.2013.03.023 23545238

[31] Ball DR , Rowlands B , Dodd MS , et al. Hyperpolarized butyrate: a metabolic probe of short chain fatty acid metabolism in the heart. Magn Reson Med. 2014;71(5):1663‐1669. 23798473 10.1002/mrm.24849 PMC4238803

[32] Bastiaansen JA , Cheng T , Lei H , Gruetter R , Comment A . Direct noninvasive estimation of myocardial tricarboxylic acid cycle flux in vivo using hyperpolarized 13C magnetic resonance. J Mol Cell Cardiol. 2015;87:129‐137. 26297113 10.1016/j.yjmcc.2015.08.012

[33] von Morze C , Bok RA , Ohliger MA , Zhu Z , Vigneron DB , Kurhanewicz J . Hyperpolarized [13C] ketobutyrate, a molecular analog of pyruvate with modified specificity for LDH isoforms. Magn Reson Med. 2016;75(5):1894‐1900. 26059096 10.1002/mrm.25716 PMC4868134

[34] Flori A , Giovannetti G , Santarelli MF , et al. Biomolecular imaging of 13C‐butyrate with dissolution‐DNP: Polarization enhancement and formulation for in vivo studies. Spectrochim Acta A Mol Biomol Spectrosc. 2018;199:153‐160. 29597071 10.1016/j.saa.2018.03.014

[35] Abdurrachim D , Teo XQ , Woo CC , et al. Cardiac metabolic modulation upon low‐carbohydrate low‐protein ketogenic diet in diabetic rats studied in vivo using hyperpolarized 13C pyruvate, butyrate and acetoacetate probes. Diabetes Obes Metab. 2019;21(4):949‐960. 30536560 10.1111/dom.13608

[36] Yoshihara HA , Bastiaansen JA , Karlsson M , Lerche MH , Comment A , Schwitter J . Detection of myocardial medium‐chain fatty acid oxidation and tricarboxylic acid cycle activity with hyperpolarized [1–13C] octanoate. NMR Biomed. 2020;33:e4243. 31904900 10.1002/nbm.4243

[37] Bastiaansen JA , Yoshihara HA , Takado Y , Gruetter R , Comment A . Hyperpolarized 13 C lactate as a substrate for in vivo metabolic studies in skeletal muscle. Metabolomics. 2014;10(5):986‐994.

[38] Takado Y , Cheng T , Bastiaansen JA , et al. Hyperpolarized 13C magnetic resonance spectroscopy reveals the rate‐limiting role of the blood–brain barrier in the cerebral uptake and metabolism of l‐lactate in vivo. ACS Chem Nerosci. 2018;9(11):2554‐2562. 10.1021/acschemneuro.8b00066 PMC6119468 29771492

[39] Hu S , Zhu M , Yoshihara HA , et al. In vivo measurement of normal rat intracellular pyruvate and lactate levels after injection of hyperpolarized [1‐13C] alanine. Magn Reson Imaging. 2011;29(8):1035‐1040. 21855243 10.1016/j.mri.2011.07.001 PMC3172390

[40] Wishart DS , Feunang YD , Marcu A , et al. HMDB 4.0: the human metabolome database for 2018. Nucleic Acids Res. 2018;46(D1):D608‐D617. 29140435 10.1093/nar/gkx1089 PMC5753273

[41] Bremmer J . Pyruvate dehydrogenase, substrate specificity and product inhibition. Eur J Biochem. 1969;8(4):535‐540. 4307688 10.1111/j.1432-1033.1969.tb00559.x

[42] Withycombe WA , Plummer DT , Wilkinson JH . Organ specificity and lactate‐dehydrogenase activity. Differential inhibition by urea and related compounds. Biochem J. 1965;94(2):384‐389. 14348198 10.1042/bj0940384 PMC1206520

[43] Wilks HM , Halsall DJ , Atkinson T , Chia WN , Clarke AR , Holbrook JJ . Designs for a broad substrate specificity keto acid dehydrogenase. Biochemistry. 1990;29(37):8587‐8591. 2271542 10.1021/bi00489a013

[44] Kim MJ , Whitesides GM . L‐Lactate dehydrogenase: substrate specificity and use as a catalyst in the synthesis of homochiral 2‐hydroxy acids. J Am Chem Soc. 1988;110(9):2959‐2964. 10.1007/BF02921743 2610514

[45] Cheng T , Capozzi A , Takado Y , Balzan R , Comment A . Over 35% liquid‐state 13 C polarization obtained via dissolution dynamic nuclear polarization at 7 T and 1 K using ubiquitous nitroxyl radicals. Phys Chem Chem Phys. 2013;15(48):20819‐20822. 24217111 10.1039/c3cp53022a

[46] Comment A , van den Brandt B , Uffmann K , et al. Design and performance of a DNP prepolarizer coupled to a rodent MRI scanner. Concepts Magn Reson Part B. 2007;31(4):255‐269.

[47] Cheng T , Mishkovsky M , Bastiaansen JA , et al. Automated transfer and injection of hyperpolarized molecules with polarization measurement prior to in vivo NMR. NMR Biomed. 2013;26(11):1582‐1588. 23893539 10.1002/nbm.2993

[48] Shaka AJ , Keeler J , Frenkiel T , Freeman RAY . An improved sequence for broadband decoupling: WALTZ‐16. J Magn Reson. 1983;52(2):335‐338.

[49] Staewen RS , Johnson AJ , Ross BD , Parrish T , Merkle H , Garwood M . 3‐D FLASH imaging using a single surface coil and a new adiabatic pulse, BIR‐4. Invest Radiol. 1990;25(5):559‐567. 2345088 10.1097/00004424-199005000-00015

[50] Adeva B , Arik E , Ahmad S , et al. Large enhancement of deuteron polarization with frequency modulated microwaves. Nucl Instrum Methods Phys Res A. 1996;372(3):339‐343.

[51] Hovav Y , Feintuch A , Vega S , Goldfarb D . Dynamic nuclear polarization using frequency modulation at 3.34 T. J Magn Reson. 2014;238:94‐105. 24333831 10.1016/j.jmr.2013.10.025

[52] Bornet A , Milani J , Vuichoud B , Linde AJP , Bodenhausen G , Jannin S . Microwave frequency modulation to enhance dissolution dynamic nuclear polarization. Chem Phys Lett. 2014;602:63‐67.

[53] Ardenkjær‐Larsen JH , Bowen S , Petersen JR , et al. Cryogen‐free dissolution dynamic nuclear polarization polarizer operating at 3.35 T, 6.70 T, and 10.1 T. Magn Reson Med. 2019;81(3):2184‐2194. 30357898 10.1002/mrm.27537

[54] Mishkovsky M , Anderson B , Karlsson M , et al. Measuring glucose cerebral metabolism in the healthy mouse using hyperpolarized 13 C magnetic resonance. Sci Rep. 2017;7(1):1‐8. 28916775 10.1038/s41598-017-12086-z PMC5601924

