# Supplementary material for: Radical‐free hyperpolarized MRI using endogenously occurring pyruvate analogues and UV‐induced nonpersistent radicals
Source: NMR Biomed. 2021 Jul 10;34(11):e4584. doi: 10.1002/nbm.4584 (PMC8518970; doi:10.1002/nbm.4584)
Supplement: Supplementary file 1 — Figure S1. Concentration calibration curve of TEMPOL in glycerol‐water (v/v 1:1) and linear fit (R2 = 0.999, red) for n = 4 data sets. Data was acquired with X‐band ESR at 77 K and subsequently used to quantify concentrations of UV‐induced radicals. Figure S2. In vivo spectra acquired immediately after injection depict the injected 13 C labeled substrate and the natural abundance 13 C resonances of the polarizing agent αkV (top) and αkB (bottom). Metabolic products are absent in these spectra. Visible resonances are [1– 13 C]butyrate, [1– 13 C]αkV, [2‐ 13 C]αkV, [1– 13 C]αkB‐hydrate, [1– 13 C]αkB and [2‐ 13 C]αkB. The resonance of [1– 13 C]αkV‐hydrate could not be detected. [file NBM-34-e4584-s001.docx]

# Supplementary Material

# Radical-free hyperpolarized MRI using endogenously-occuring pyruvate analogues and UV-induced nonpersistent radicals

Claudia C Zanella^1^, Andrea Capozzi^1^, Hikari AI Yoshihara^1^, Alice Radaelli^1^, Adèle LC Mackowiak^2^, Lionel P Arn^2^, Rolf Gruetter^1^, Jessica AM Bastiaansen^2^

^1^ Laboratory of Functional and Metabolic Imaging, EPFL, Lausanne, Switzerland.

^2^ Department of Diagnostic and Interventional Radiology, CHUV, Lausanne, Switzerland.

# Supplementary methods

## Empirical procedure for optimizing sample formulation

The sample formulation was empirically optimized to obtain 40 mM of radical concentration in beads, which has previously shown to be well-suited for nitroxyl radicals^1,2^, with simultaneous satisfactory bead consistency. It is important to point out that samples were not specifically optimized to maximize radical yield, to minimize precursor concentration, to minimize the UV-irradiation time or to maximize achievable polarization levels.

In a first step, the maximum unlabeled Glc or BA concentration in glycerol-water was determined such that the solution created glassy beads upon freezing droplets of it in liquid nitrogen (according to a visual assessment). This solution acted as starting point for the next step.

In the second step, increasing volumes of radical precursors were added to reach our target value of 40 mM while ensuring that a maximum of 10% of the irradiated beads pulverized during UV-irradiation. If beads would not match the previous condition, glycerol-water was added in an iterative process until one possible mix yielding robust beads of 40 mM radical concentration was found. The UV-irradiation time of 200 s (for 6 ul beads) was chosen to avoid bulk pulverization (observed for 320 s of UV-irradiation) and to achieve the range of plateauing radical concentration (above 180 s UV-irradiation.

# Supplementary Figures


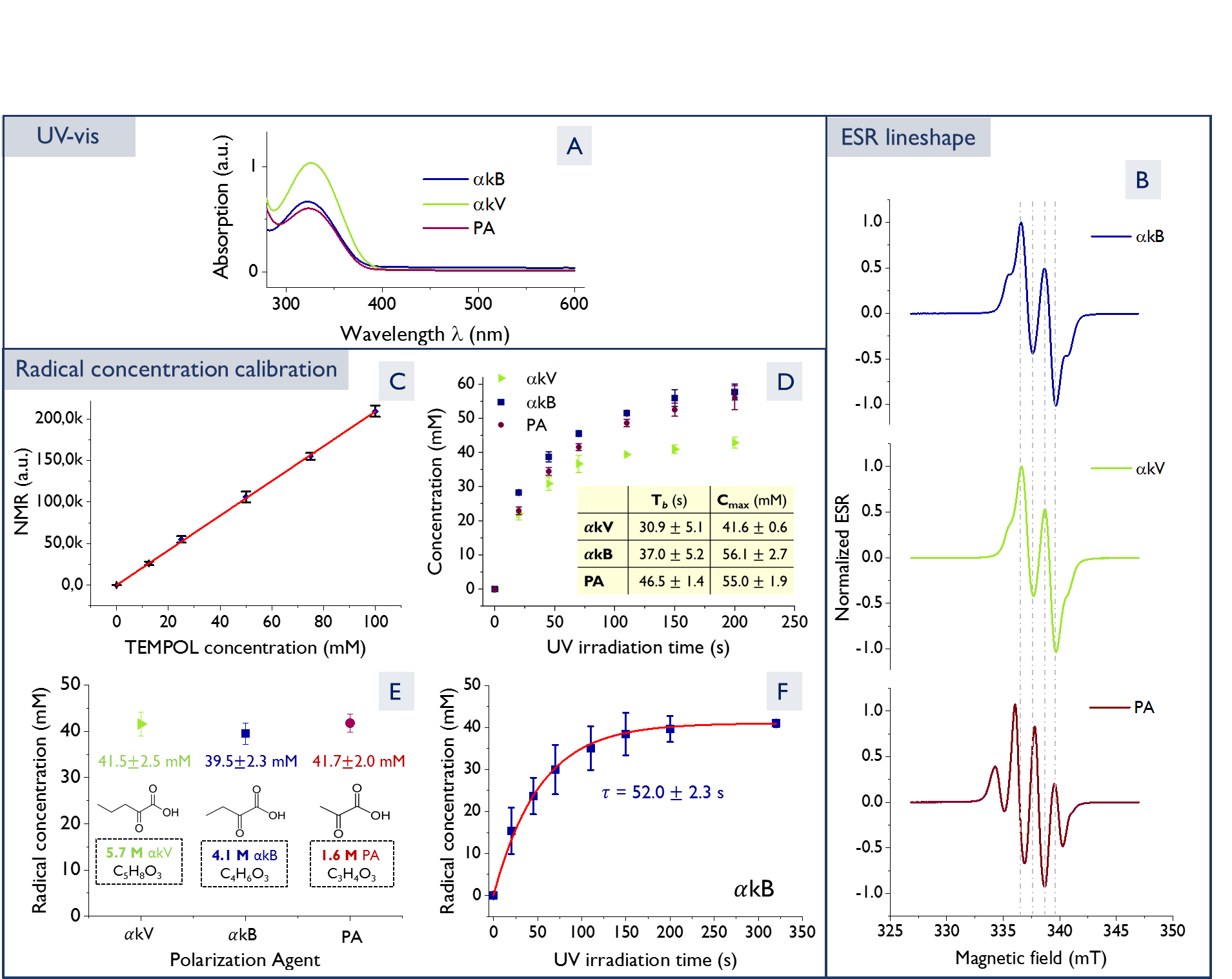


## Figure S1

Concentration calibration curve of TEMPOL in glycerol-water (v/v 1:1) and linear fit (R^2^ = 0.999, red) for n = 4 data sets. Data was acquired with X-band ESR at 77 K and subsequently used to quantify concentrations of UV-induced radicals.

## Figure S2

*In vivo* spectra acquired immediately after injection depict the injected ^13^C labeled substrate and the natural abundance ^13^C resonances of the polarizing agent$\alpha$kV (top) and $\alpha$kB (bottom). Metabolic products are absent in these spectra. Visible resonances are [1-^13^C]butyrate, [1-^13^C]$\alpha$kV, [2-^13^C]$\alpha$kV, [1-^13^C]$\alpha$kB-hydrate, [1-^13^C]$\alpha$kB and [2-^13^C]$\alpha$kB. The resonance of [1-^13^C]$\alpha$kV-hydrate could not be detected.

# REFERENCES

1. Bastiaansen, J. A., Yoshihara, H. A., Takado, Y., Gruetter, R. & Comment, A. Hyperpolarized 13 C lactate as a substrate for in vivo metabolic studies in skeletal muscle. *Metabolomics* **10**, 986–994 (2014).

2. Cheng, T., Capozzi, A., Takado, Y., Balzan, R. & Comment, A. Over 35% liquid-state 13 C polarization obtained via dissolution dynamic nuclear polarization at 7 T and 1 K using ubiquitous nitroxyl radicals. *Phys. Chem. Chem. Phys.* **15**, 20819–20822 (2013).
